# The effects of Desflurane and Sevoflurane on Nesfatin-1 levels in laparoscopic Cholecystectomy: a randomized controlled trial

**DOI:** 10.1186/s12871-018-0484-x

**Published:** 2018-02-16

**Authors:** A. T. D. Ozcan, C. B. Altin, S. Erdogan, M. Ergin, A. Çiftçi, H. Kara, S. M. Aksoy, A. But

**Affiliations:** 1 Atatürk Training and Research Hospital, Anesthesiology and Reanimation Department, Atatürk Training and Research Hospital, Bilkent, Ankara, Turkey; 2 Yıldırım Beyazıt University Anesthesiology and Reanimation Department, Ankara, Turkey; 3 Atatürk Training and Research Hospital, Biochemistry Department, Ankara, Turkey; 4 Yıldırım Beyazıt University Pharmacology Department, Ankara, Turkey

**Keywords:** Desflurane, Inhaler anesthetic agent, Sevoflurane, Volatile anesthetics, Nesfatin-1, Laparoscopic abdominal surgery

## Abstract

**Background:**

Nesfatin-1 is involved in cardiovascular regulation, stress-related responses. The objective of this study is to investigate the impact of volatile anesthetics on Nesfatin-1 levels.

**Method:**

Fourty-two patients aged 30–65 years with the American Society Anesthesiology (ASA) Class I-II who were scheduled for laparoscopic cholecystectomy were included in the study Patients were randomized into two group; desflurane administered group (Group I, *n* = 21) and sevoflurane administered group (Group II, n = 21). For anesthesia maintenance, the patients received 6% desflurane or 2% sevoflurane in 40% O2 and 60% air. The patient’s heart rate (HR), mean, systolic and diastolic arterial pressures (MAP, SAP, DAP), peripheral O2 saturation (SpO2) were monitored and recorded before induction, after induction, after intubation, and during extubation. Blood samples were collected before induction (T1), and after extubation when aldrete score was 10 (T2).

**Results:**

Demographic data were similar between the groups. The preoperative levels of nesfatin were similar in the two groups (*p* = 0.715). In desflurane group, post-operative nesfatin levels were similar compared to preoperative levels (*p* = 0.073). In sevoflurane group, post-operative nesfatin levels were similar (*p* = 0.131). The nesfatin levels (postoperative vs preoperative) were similar between the groups (*p* = 0.900).

**Conclusion:**

In conclusion, this study results suggest that nesfatin-1 levels are not affected by the use of sevoflurane or desflurane in patients undergoing laparoscopic cholecystectomy.

**Trial registration:**

Australian New Zealand Clinical Trials Registry, ACTRN12617001023347, retrospectively registered on 17 July 2017.

## Background

Nesfatin-1 was discovered in 2006 by Oh-I et al. [1] as an 82-amino acid (aa) polypeptide derived from the calcium and DNA-binding protein, nucleobindin 2 (NUCB2). Human studies have shown a circulating NUCB2/nesfatin-1 concentration of 30 pmol/L [2]. Nesfatin-1 is an anorexigenic, energy-regulating peptide expressed in not only central, but also peripheral tissues with pleiotropic effects [3].

In addition to its central expression and actions, NUCB2/nesfatin-1 has been subsequently described to be predominantly expressed in the periphery and to exert several peripheral effects in adipose tissue, gastric mucosa, endocrine pancreatic beta cells, and the testis [2]. Nesfatin-1 reduces gastric emptying and motility after brain injection [4], and it is also involved in the regulation of cardiovascular functions by stimulating sympathetic nerve activity [5].

NUCB2/ nesfatin-1 that has a regulatory role in cardiac functions and expression from cardiomyocytes has been showed in rats and humans [6]. Nesfatin-1 is involved in other important processes, including cardiovascular regulation, stress-related responses [3, 7]. Gastric transit and oral intake are delayed by the possible effect of the nesfatin-1 after the abdominal surgery. [8].

Today, laparoscopic cholecystectomy has been increasingly used. Endocrine markers were elevated because of the stress response in experimental animals and humans undergoing abdominal surgery [8]. İt has been identified by different studies that acute stress increases the central level of nesfatin-1 [9, 10]. However, according to Yoshida et al. acute stress does not influence plasma level of nesfatin-1 [11].

In the present study, we aimed to analyze the changes in nesfatin-1 levels in patients undergoing laparoscopic cholecystectomy based on postoperative surgical stress responses, and to compare the effect of two different volatile anesthetics on plasmatic levels of nesfatin-1.

## Methods

A written informed consent was obtained from each patient. The study protocol was approved by the Yıldırım Beyazid University Clinical Research Ethics Committee (Number: 26,379,996/259). The study was conducted in accordance with the principles of the Declaration of Helsinki.

Forty-two patients with the American Society Anesthesiology (ASA) Class I-II who were scheduled for laparoscopic cholecystectomy between 01.12.2016 and 01.01.2017 were included in the study.

Prior to anesthesia induction, patients were randomly assigned to two groups using a sealed envelope system. Heart rate (HR), mean, systolic and diastolic arterial pressures (MAP, SAP, DAP), peripheral O2 saturation (SpO_2_) of the patients who were taken to the operation were monitored. A 20 gauge catheter was used to enable intravenous access, and 0.9% sodium chloride (5–10 mL/kg/h) was infused. To induce anesthesia, the patients were given 1 mg of lidocaine (Aritmal, 2%, Osel), 1 μg/kg of remifentanil (Ultiva, 5 mg, GlaxoSmithKline), 8 mg/kg of thiopental sodium, and 0.6 mg/kg of rocuronium bromide intravenously. After a three-min preoxygenation with 100% O_2_ administration through a face mask, orotracheal intubation was performed when sufficient muscle relaxation was observed. Then, patients were ventilated with a Dräger anesthesia instrument (Luebeck, Germany), with tidal volume set to 8 ml kg^− 1^ and frequency set to 12/min. Soda lime (Sorbo-lime, Berkim, Turkey) was used as a CO_2_ absorbant. For anesthesia maintenance, the patients received 6% desflurane or 2% sevoflurane in 40% O2 and 60% air, according their randomization. 8 ml kg^− 1^ tidal volume (TV) ventilation with 5–7 cmH_2_O PEEP were applied to the patients during surgery.

Patients in both groups received remifentanil infusion (0.25 μg/kg/min) to maintain anesthesia. At the end of the surgery, all patients received 0.5 mg atropine and 1.5 mg neostigmine for decurarization. In all groups, patients received 0.5 mg atropine when heart rate was < 40; 10 mg of ephedrine when MAP was < 50, and the remifentanil infusion dose was lowered. Sevoflurane and desflurane concentrations weren’t changed during the anesthesia maintenance. Patients received intravenous tramadol (1 mg/kg) and metoclopramide (10 mg) 30 min before the end of surgery.

Hemodynamic and respiratory parameters (SAP, DAP, MAP, HR, SpO_2_, etCO_2_) were recorded before induction, after induction, after intubation, and during extubation. Blood samples were collected before induction (T1), and after extubation when aldrete score was 10 (T2). Blood samples were collected into aprotinin and EDTA-containing tubes. Samples were centrifuged at 3000 rpm, and plasma samples were aliquoted in polypropylene tubes, and stored at − 80 C until further analysis. Five cc of venous blood samples were used to analyze nesfatin-1 levels, by using a commercial ELISA kit according to the vendor’s instructions. (Ray- Biotech, Norcross, GA, USA; catalogue no. EIANES-1).

### Statistical analysis

Data analysis was performed by using IBM SPSS Statistics version 17.0 software (SPSS Inc., Chicago, IL, USA). Shapiro Wilk test was used to determine whether the distributions of continuous variables were normal or not. While categorical data were shown in number of cases and percentages, descriptive statistics for continuous variables were expressed in mean ± SD or median (min-max), where applicable. Categorical data were analyzed by the continuity corrected chi-square or Fisher’s exact test, where appropriate. The mean differences between the groups were compared by Student’s t test, while the Mann-Whitney U test was used to compare the mean ranks. Wilcoxon sign-rank test was used to assess the statistical significance of the differences between pre- and post-operative nesfatin levels. A *p* value less than 0.05 was considered statistically significant.

### Sample size estimation

The primary aim of this study was to compare by means of differences in nesfatin measurements between the groups. A total sample size of 42 (21 in each group) was required to detect at least 30.6-U difference with a power of 80% at 5% significance level. The difference of 30.6 was taken from both pilot study and clinical experience. The sample size estimation was performed by using G*Power 3.0.10 software (Franz Faul, Universitat Kiel, Germany).

## Results

There were no significant differences in the mean age, sex, ASA stage, mean height, mean body weight, body mass index (BMI), and distribution of comorbidities between desflurane and sevoflurane groups (*p* > 0.05) (Table 1).

**Table 1:** Demographic and clinical features of groups

Parameters	Desflurane (*n* = 21)	Sevoflurane (*n* = 21)	*p*-value
Age (years)	49.6 ± 9.3	49.8 ± 10.9	0.943^a^
Sex			0.306^b^
Male	4 (19.0%)	8 (38.1%)	
Female	17 (81.0%)	13 (61.9%)	
ASA			1.000^b^
I	11 (52.4%)	10 (47.6%)	
II	10 (47.6%)	11 (52.4%)	
Height (m)	1.64 ± 0.10	1.66 ± 0.10	0.566^a^
Body weight (kg)	84.0 ± 17.9	79.3 ± 13.2	0.366^a^
Body-mass index (kg/m^2^)	31.2 ± 5.9	28.9 ± 5.2	0.210^a^
Comorbid diseases	10 (47.6%)	11 (52.4%)	1.000^b^
* DM*	1 (4.8%)	6 (28.6%)	0.093^c^
* HT*	7 (33.3%)	6 (28.6%)	1.000^b^
Asthma	2 (9.5%)	4 (19.0%)	0.663^c^
Others	4 (19.0%)	3 (14.3%)	1.000^c^

^a^ Student’s t test, ^b^ Continuity corrected chi-square test, ^c^Fisher’s exact test. Others: thyroid, hematological, neurological and rheumatological diseases

There was no significant difference in preoperative nesfatin levels between desflurane and sevoflurane groups (*p* = 0.715).

**Fig. 1:**
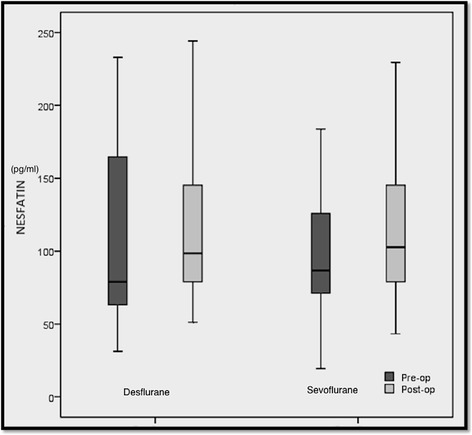
Preoperative and postoperative nesfatin-1 levels (pg/ml) at each groups.In desflurane group, post-operative nesfatin levels were similar compared to preoperative levels (*p* = 0.073). There was no significant difference in post-operative nesfatin levels between desflurane and sevoflurane groups (*p* = 0.900). (Figure 1)

In desflurane group, post-operative nesfatin levels were similar compared to preoperative levels (*p* = 0.073). Post-operative nesfatin levels in sevoflurane group were similar (*p* = 0.131). There was no significant difference in post-operative nesfatin levels between desflurane and sevoflurane groups (*p* = 0.900). The nesfatin levels (postoperative vs preoperative) were similar between the groups (p = 0.900). (Table 2) (Fig. 1).

**Table 2:** Pre- and postoperative nesfatin levels

	Preoperative Nesfatin Levels (pg/ml)	Postoperative Nesfatin Levels (pg/ml)	*p*-value ^a^	Change
Desflurane	78.9 (59.1–168.4)	98.6 (78.9–147.2)	0.073	15.8 (−13.6–41.2)
Sevoflurane	86.8 (71.0–127.9)	102.5 (77.0–145.7)	0.131	3.9 (−9.9–39.3)
*p*-value^b^	0.715	0.900		0.900

^a^Comparison of pre- and postoperative nesfatin levels within the groups, Wilcoxon sign-rank test, ^b^Comparison between the groups, Mann-Whitney U test

There were no significant differences in pre-intubation, post-intubation, intraoperative 15, intraoperative 30, and post-extubation SAP, DAP, MAP, heart rate and SpO_2_ between the groups. There were no significant differences in post-intubation, intraoperative 15. min, intraoperative 30.min EtCo_2_ measurements between the groups (Table 3).

**Table 3:** Comparision of groups based on hemodynamic measurements within a single observation period

	Desflurane	Sevoflurane	*p*-value ^a^
SAP (mmHg)			
pre-intubation	149.88 ± 19.76	145.42 ± 19.09	0.496
post-intubation	121.12 ± 26.89	133.00 ± 21.16	0.148
intraop15	122.18 ± 22.70	122.89 ± 18.94	0.918
intraop30	118.88 ± 15.07	122.21 ± 18.18	0.556
post-extubation	150.71 ± 20.44	155.63 ± 23.77	0.512
DAP (mmHg)			
pre-intubation	88.71 ± 10.48	88.58 ± 10.52	0.971
post-intubation	74.94 ± 17.32	84.68 ± 14.69	0.077
intraop15	82.29 ± 16.63	78.89 ± 13.01	0.497
intraop30	73.00 ± 20.14	73.63 ± 20.64	0.927
post-extubation	95.06 ± 11.37	93.68 ± 13.01	0.739
MAP(mmHg)			
pre-intubation	111.56 ± 10.75	107.47 ± 14.83	0.374
post-intubation	86.94 ± 20.23	99.24 ± 17.84	0.073
intraop15	94.44 ± 20.44	93.29 ± 14.98	0.855
intraop30	90.56 ± 12.30	91.94 ± 15.05	0.776
post-extubation	115.31 ± 16.63	118.06 ± 16.22	0.634
HR(heart rate per minute)			
pre-intubation	87.76 ± 18.38	80.32 ± 12.95	0.165
post-intubation	84.00 ± 21.04	79.32 ± 12.61	0.418
intraop15	68.18 ± 13.68	67.47 ± 11.84	0.870
intraop30	65.82 ± 12.97	63.47 ± 10.07	0.545
post-extubation	92.35 ± 14.78	84.21 ± 13.72	0.096
SpO_2_(%)			
pre-intubation	97.76 ± 1.60	98.26 ± 1.52	0.345
post-intubation	98.59 ± 1.80	99.26 ± 0.81	0.170
intraop15	98.76 ± 1.09	98.68 ± 1.67	0.867
intraop30	98.65 ± 1.06	98.95 ± 1.08	0.406
post-extubation	98.82 ± 1.78	98.68 ± 1.97	0.826
EtCo_2_(mmHg)			
post-intubation	34.00 ± 4.81	33.52 ± 4.65	0.749
intraop15	33.75 ± 2.57	32.90 ± 3.27	0.365
intraop30	35.65 ± 3.05	34.71 ± 2.97	0.326

^a^Student’s t test

For all patients, age and body weight were not significantly correlated with the change in nesfatin levels (*p* = 0.249 and *p* = 0.081, respectively). Similarly, the variation in nesfatin levels was similar between female and male patients (*p* = 0.731). The increase in nesfatin levels was similar between the diabetic group and the non-diabetic group (*p* = 0.974). The increase in nesfatin levels was also similar between the hypertensive group and the non-hypertensive group (*p* = 0.872).

There was no statistically significant difference between pre- and post-op nesfatin levels in diabetic patients (*n* = 7) and non-diabetic patients (*n* = 35) (*p* = 0.303 and *p* = 0.370). Postoperative nesfatin levels compare to preoperative levels have increased in statistically similar manner in diabetic patients and nondiabetic patients(*p* = 0,974). Moreover no correlation has been observed between nesfatin levels and duration of surgery.

The horizontal lines in the middle of each box indicates the median Nesfatin-1 levels, while the top and bottom borders of the box mark the 25th and 75th percentiles, respectively. The whiskers above and below the box mark indicates the maximum and minimum levels.

## Discussion

The major finding of this study is, that nesfatin-1 levels are unaffected by laparoscopic cholecystectomy and remain unchanged by sevoflurane and desflurane. Neurohumoral, immunological, and metabolic pathways are activated through surgery, and these events depend on the duration of surgery, intensity of postoperative pain, degree of blood loss, and type of surgery [12].

Regarding the effect of inhaled agents on surgical stress response, desflurane has a lower cortisol and ACTH response, compared to sevoflurane; however, desflurane is known to affect catecholamine levels more, compared to sevoflurane, and also enhances catecholamine secretion [13]. Therefore, sevoflurane may be more useful in patients with cardiovascular instability, hypertension, and cardiac pathology.

Nesfatin-1 is involved in the regulation of cardiovascular functions through stimulation of sympathetic nerves [14]. Feijoo-Bandin et al. [6] demonstrated the presence of NUCB2/nesfatin-1 in both rat and human cardiomyocytes, which support the hypothesis that nesfatin-1 may have a regulatory role in cardiac functions. Subcutaneous administration of nesfatin-1 to the rats led to a prominent increase in MAP without affecting the heart rate, suggesting a beta-adrenergic effect of nesfatin-1 [11]. While cardiac and renal beta-adrenergic activation have an important role in the regulation of systemic blood pressure, hypothalamic nesfatin is considered to be the mainstay in this process [15].

Similar to MAP, nesfatin-1 signaling is considered to be an important factor in modulation of cardiovascular responses [16]. In this study, there was no significant difference in the hemodynamic parameters between the patient groups.

However, we did not identify a correlation between nesfatin levels and BMI in this study. Nesfatin-1 is considered as an important hormone for regulating body weight in humans. Efforts to identify the correlation between BMI and nesfatin levels have provided contradictory results. Some of the previous studies have suggested a negative correlation between BMI and nesfatin levels [17, 18] whereas others have suggested a positive correlation between these variables [19, 20].

In another study, intracerebroventricular injection of nesfatin-1 was found to increase anxiety- and fear-associated behavior in rats [21]. Central nesfatin-1 plays a role in anxiety [22], and has been linked to response to various types of stress [23]. Goebel et al. suggested that nesfatin plays a regulatory role in stress condition [9].

In mice, nesfatin-1 precursor proteins are expressed in brain regions involved in stress response and cognitive functions [24].

On the other hand, nesfatin-1-mediated regulation of stress and mood may be associated with a sex-specific pathway [14]. Elevated nesfatin-1 levels have been linked to age [25], sex [26], and anxiety [27]. However, there are contradictory reports on positive/negative correlation between nesfatin-1 levels and female sex [28, 29]. Another study has shown that circulating nesfatin-1 levels are associated with anxiety in obese female patients [2] and female patients with anorexia nervosa [26]. On the other hand, an inverse correlation has been identified in obese male patients [30]. In this study, we did not identify any correlation among nesfatin-1 levels, sex and age.

Comparison of rats who underwent surgery under isoflurane anesthesia and who did not undergo surgery showed nesfatin-1 secretion, particularly in the forebrain, from neuroendocrine nerves (SON and PVN) and anterior parvocellular neurons. The central level of nesfatin-1 can be increased by acute stress; on the other hand, the plasma levels of nesfatin-1 are not influenced by acute stress. A recent study has shown that chronic stress may increase the plasma level of nesfatin-1 [11].

Stress limited to 30 min increased nesfatin-1 secretion. Behavioral, endocrine, and autonomic responses to stress should be evaluated to understand the place of nesfatin-1 in physiological stress response. In another study, 30-min stress was found to cause nesfatin secretion from pontine (LC)/medullary nuclei most raphe pallidus neurons, SON neurons, and, to a smaller extent, neurons in the magno- and parvocellular subdivisions of the PVN, similar to activation of catecholaminergic neurons [9].

Extracellular norepinephrine levels are elevated by restraint, tail shock, auditory and hypotensive stress, in LC terminal regions [31] and elevated levels of Fos mRNA and protein in the LC are induced in response to abdominal surgery, restraint, shock, hypotension, swim force, immune challenge, water avoidance stress and social stress [32–34]. In Another research, it was finded out that the abdominal surgery activates the nesfatin-1-ir neurons in the LC combined with LC-arising projections to CRF-containing neurons in the PVN. And then release of brain CRF lend to the reduce of gastric motor function so by this way postoperative ileus after abdominal surgery can be developed [4].

Nesfatin-1-producing neurons become activated in response to acute limited stress. This, in turn, modulates bowel functions [10]. Nesfatin-1 may also play a role in important metabolism-related stress adaptive responses, such as suppression of food intake.

Intracerebroventricular administration of nesfatin leads to a dose-dependent delay in gastric emptying. Explorative abdominal surgery was indicated as hypothalamus and medullary neurons were activated, which regulates postoperative gastric ileus and digestion functions [35].

The results of the studies analyzing stress response to surgery have revealed elevated postoperative nesfatin-1 levels, and reduced bowel motility. We could not detect a significant increase in association with the response to surgical stress in nesfatin levels. In future studies, we will analyze nesfatin levels in patients with reduced anxiety due to intravenous hypnotic agents, and investigate the effects of premedication on bowel motility and discharge from hospital.

## Conclusion

In conclusion, this study results suggest that nesfatin-1 levels are not affected by the use of sevoflurane or desflurane in patients undergoing laparoscopic cholecystectomy.

## Abbreviations

ARC: Arcuate nucleus
ASA: American Society Anesthesiology
DAP: Diastolic arterial pressure
DMNX: Dorsal motor nucleus of the vagus
etCO2: End tidal carbon dioxide
HR: Heart rate
LC: Locus ceruleus.
MAP: Mean arterial pressure
NTS: Tractus solitarius
NUCB2: Nucleobindin 2
PVN: Paraventricular nucleus
SAP: Systolic arterial pressure
SON: Supraoptic nucleus
SpO2: Oxigen saturation
